# Prediagnosis obesity and secondary primary cancer risk in female cancer survivors: A national cohort study

**DOI:** 10.1002/cam4.1959

**Published:** 2019-01-16

**Authors:** So‐Youn Jung, Young Ae Kim, Minkyung Jo, Sang Min Park, Young‐Joo Won, Haryeom Ghang, Sun‐Young Kong, Kyu‐Won Jung, Eun Sook Lee

**Affiliations:** ^1^ Center for Breast Cancer Research Institute and Hospital National Cancer Center Goyang Korea; ^2^ Cancer Policy Branch National Cancer Control Institute National Cancer Center Goyang Korea; ^3^ Department of Biomedical Sciences Seoul National University College of Medicine Seoul Korea; ^4^ Department of Family Medicine Seoul National University College of Medicine Seoul Korea; ^5^ Cancer Registration and Statistics Branch National Cancer Control Institute National Cancer Center Goyang Korea; ^6^ Health Insurance Policy Research Institute National Health Insurance Service Gangwon‐do Korea; ^7^ Department of Cancer Biomedical Science National Cancer Center Graduate School of Cancer Science and Policy Goyang Korea; ^8^ Department of Laboratory Medicine Research Institute and Hospital National Cancer Center Goyang Korea; ^9^ Translational Cancer Research Branch Research Institute and Hospital National Cancer Center Goyang Korea

**Keywords:** body mass index, cancer survivor, female, Korea, obesity, second primary cancer

## Abstract

**Background:**

This study evaluated the effects of body mass index (BMI) before the diagnosis of the first primary cancer on the development of secondary primary cancers (SPCs) in female cancer survivors.

**Methods:**

This study population included 146 377 Korean female cancer survivors whose first primary cancer was diagnosed between 2002 and 2010. The incidence of SPCs was evaluated throughout follow‐up until December 2011. We used Cox proportional hazards models to calculate the hazard ratios of SPCs with prediagnosis BMI and compared it to those of first cancers in the general population.

**Results:**

After 565 877 person‐years of follow‐up, 2222 patients with SPC were observed. The higher BMI was more in female cancer survivors than in general population. The age‐standardized incidence rate of cancer in cancer survivors was 2.02 times higher than that of the general population. There were positive linear trends between prediagnosis BMI and risk of overall, colorectal, ovary, thyroid, and obesity‐related SPCs. In addition, the BMI‐SPC risk association was statistically significant in female cancer survivors without smoking history (*P*_trend* *_= 0.001) and with a localized first primary cancer (*P*_trend* *_= 0.014). However, the magnitude of the BMI‐SPC risk association was similar to that for first cancers in the general population (*P*_heterogeneity _= 0.403 in BMI ≥ 30.0 kg/m^2^).

**Conclusions:**

In female cancer survivors, prediagnosis obesity was a risk factor for overall, individual, and obesity‐related SPCs. However, the magnitude of the BMI‐SPC risk association was similar to that for first cancers in the general population.

## Abstract 2

In this Korean national cohort study, which included 146 377 Korean female cancer survivors whose first primary cancer was diagnosed between 2002 and 2010, the age‐standardized incidence rate of cancer in cancer survivors was higher than that of the general population. However, the magnitude of the body mass index (BMI)‐SPC risk association was similar to that for first cancers in the general population.

## 1 INTRODUCTION

The increasing number of cancer survivors has resulted from early cancer detection and advances in cancer treatment.^1^ However, many cancer survivors also have an increased risk of secondary primary cancers (SPCs), which might be greater than the risk of primary cancers in the general population.^2^, ^3^, ^4^, ^5^, ^6^, ^7^ A recent study reported that mortality due to SPCs was higher than mortality due to first primary cancer.^8^ SPCs have been associated with genetic susceptibility,^9^, ^10^ the carcinogenic effects of cancer treatment,^11^ and the influence of behavioral risk factors such as smoking and alcohol intake.^12^, ^13^, ^14^, ^15^, ^16^

Obesity is a well‐known risk factor for cancer in the general population including cancers of the colon, lower esophagus, kidney, gallbladder, breast, and endometrium.^17^, ^18^, ^19^, ^20^, ^21^, ^22^ In addition, several studies have reported the association between obesity and increased SPC risk at specific sites such as breast^23^ and colorectum^24^ Although the mechanisms of the linkage between obesity and cancer risk have not been fully elucidated, hormones, including sex hormones, have been associated with increased cancer risk^21^, ^25^ Therefore, it would be relevant to evaluate the correlation between obesity and cancer risk separately according to sex. Previously, we reported that obesity was associated with increased primary cancer risk^26^ and SPC risk^6^, ^27^ in Korean male survivors. These studies demonstrated that male cancer survivors who had a higher prediagnosis BMI had an increased risk of subsequent overall SPCs and that the magnitude of the association between obesity and SPC risk was stronger than that of first primary cancer risk.^27^ However, there was no study for evaluating it in female cancer survivors.

This study evaluated the association between prediagnosis BMI and SPC risk in female cancer survivors by analyzing merged data from the Korean National Health Insurance Service (NHIS) and Korea Cental Cancer Registry (KCCR). We also compared the risk of SPC in obese female cancer survivors vs the risk of first primary cancers in the general population of women.

## 2 METHODS

### 2.1 Study population

This study included Korean female cancer survivors, who got health examinations by the NHIS before their first cancer diagnosis during January 2002 and December 2010. As previously mentioned,^27^ the Korean NHIS is the only public health insurer in Korea, and it provides biennial health examinations, including height and weight measurements and behavioral surveys, in which 68.2% of Koreans have been participated in 2010.^28^ The KCCR is a population‐based national cancer registry that includes information on more than 95% of patients with newly diagnosed cancer in Korea.^29^, ^30^ This study was approved by the Institutional Review Board of the National Cancer Center, Korea (NCC2015‐2017) and was exempt from the requirement of informed consent because the information in these datasets had been de‐identified.

We identified 11 175 133 women who were 18 years of age or older and used the Korean NHIS at least once between 2002 and 2010. We then excluded female survivors without information on prediagnosis BMI (N* *=* *8295), who were diagnosed with thyroid cancer as the primary cancer (N* *=* *57 881) and who were dying or diagnosed with their first incidence of cancer within 1 year after baseline NHIS examination (N* *=* *146 572). Among the remaining 10 962 385 women, we selected 146 377 female cancer survivors with their first primary cancer diagnosed between 2002 and 2010. The primary endpoint of this study was a newly diagnosed SPC, defined as a cancer with a different topology using the International Classification of Diseases, Tenth Revision [ICD‐10] at least 2 months later than the first primary cancer.

### 2.2 Assessment of prediagnosis exposure and covariates

We collected health‐related information from self‐reported questionnaires including previous medical history, current health status, smoking, alcohol intake, diet, exercise, and family history. We obtained medical comorbidities, place of residence, and insurance level for evaluating about socioeconomic status using the NHIS database.

Prediagnosis information including height and weight was collected from the one follow‐up cycle data before the primary cancer diagnosis. Height and weight were measured directly by trained persons. We calculated prediagnosis BMI and divided them by WHO criteria for Asian populations. A BMI with 18.6‐22.9 kg/m^2^ was categorized as normal, 23.0‐24.9 as overweight, 25.0‐29.9 as obese, and ≥30 as severely obese.^31^

### 2.3 Statistical analysis

For each individual, the accumulated person‐years of risk were calculated from the date of diagnosis of the first primary cancer to the date of diagnosis of a SPC, death, or last follow‐up (31 December 2011), whichever came first. For comparing cancer incidence between female cancer survivors and the general women population, age‐standardized incidence rates (IRs) were estimated using a direct standardization method. We used Cox proportional hazards analysis for calculating age‐adjusted and multivariable‐adjusted hazard ratios (aHRs) for SPC development related to prediagnosis BMI. We performed the tests for trends using median values for BMI.

In the additional sensitivity analyses, we excluded individuals diagnosed with simultaneous primary cancers within 2 years of the first cancer diagnosis for minimizing detection bias. We also analyzed data adjusted for follow‐up time. We also stratified associations according to age at first cancer diagnosis, stage of first primary cancer (2006‐2010), smoking, location of residence, insurance level, and disability status. In addition, we assessed the BMI‐SPC risk association in survivors of an obesity‐related cancer. Here, obesity‐related cancers were defined as previously reported, dose‐response cancers with obesity (esophageal, colorectal, pancreas, and renal cancer).^24^, ^32^ In addition, obesity‐related female cancers also were defined as obesity‐related cancers, breast cancer, and endometrial cancer.

We also compared the strength of the association between BMI and risk of cancer in female cancer survivors vs general population using Cox proportional analysis. For the evaluation of first primary cancer risk, 10 816 008 women in general population who were seen by the Korean NHIS during 2002‐2010 were included and exposure variables were collected from the first health examination that occurred in the same periods. Follow‐up duration was from the date of the first health examination to the date of diagnosis of a first primary cancer, death, or last day of 2011, whichever came first. We calculated *P* values for heterogeneity using the Q statistic. To evaluate the potential impact of death as a competing risk, we used the Poisson regression method of Fine and Gray.^33^ We used SAS for statistical analysis (version 9.3; SAS Institute, Cary, NC).

## 3 RESULTS

### 3.1 Clinical characteristics of the study population

Table 1 shows the clinical characteristics of the study population. The mean age of female cancer survivors and the total population at baseline was 56.7 years and 45.5 years, respectively. Although mean BMI was similar in both groups (23.9 in cancer survivors vs 23.0 in total population), the proportions of the obese population (BMI ≥ 25.0 kg/m^2^) were 35.2% (30.93% with 25.0‐29.9 kg/m^2^, 4.24% with ≥30.0 kg/m^2^) for cancer survivors vs 26.1% (22.83% with 25.0‐29.9 kg/m^2^, 3.29% with ≥30.0 kg/m^2^) for the total population.

**Table 1:** Descriptive characteristics of the study population (2002‐2010)

Characteristic	Cancer survivors, prediagnosis (N = 146 377)			Total cohort, starting year (N = 10 962 385)		
	N	%	Person‐years	N	%	Person‐years
Mean age at inclusion, years (SD)	56.67	13.20		45.48	15.27	
Body mass index, kg/m^2^						
Mean (SD)	23.92	(3.28)		23.03	(3.37)	
<18.5	4958	3.39	19168.65	711 713	6.49	3356729.01
18.6‐22.9	54 277	37.08	210208.66	5 109 301	46.61	24053918.48
23‐24.9	35 664	24.36	137567.21	2 277 630	20.78	10911422.97
25‐29.9	45 268	30.93	175024.32	2 503 144	22.83	11938694.03
≥30	6210	4.24	23907.60	360 597	3.29	1576156.44
Smoking status						
Never	133 520	94.37	516219.76	10 034 872	94.02	47588054.11
Former	1970	1.39	7493.62	197 109	1.85	827942.72
Current	5999	4.24	22645.85	440 605	4.13	1760678.45
Physical activity, times/wk						
None	94 526	66.6	367694.19	6 959 224	65.09	33396605.00
1‐2	22 934	16.16	88400.65	2 027 026	18.96	9171776.37
3‐4	10 792	7.60	40465.94	914 394	8.55	3993948.33
5‐6	3397	2.39	12686.45	288 919	2.70	1160115.76
7	10 291	7.25	39330.54	502 092	4.70	2622259.12
Alcohol consumption, drinks/wk						
None	116 889	82.55	452813.03	7 532 414	71.59	36524753.81
2	22 061	15.58	86591.28	2 702 027	25.68	12536983.25
3‐4	1543	1.09	5771.36	210 190	2.00	742838.12
5	1106	0.78	4193.93	76 747	0.73	314632.09
Fasting serum glucose, mg/dL						
<100	122 143	83.65	475212.00	9 789 904	89.42	46427368.82
100‐125	12 787	8.76	48081.44	677 834	6.19	3125487.46
126‐139	3412	2.34	12706.44	161 958	1.48	724556.68
≥140	7672	5.25	28357.98	318 459	2.91	1469428.83
Fasting serum cholesterol, mg/dL						
<200	76 163	52.17	293855.24	6 718 084	61.37	31590923.91
200‐239	48 548	33.25	188448.25	3 065 740	28.01	14602741.86
≥240	21 293	14.58	82 086.69	1 162 189	10.62	5547547.08
Presence of disability						
No disability	136 534	93.30	524107.63			
Disability	9803	6.70	41617.86			
Diabetes	30 499	20.84	119541.19			
Dyslipidemia	37 024	25.3	151566.46			
Osteoporosis	3686	2.52	15076.51			
Heart disease	7616	5.2	29850.23			
Liver disease	35 328	24.14	137972.55			
Cerebrovascular disease	13 996	9.56	57541.04			
SEER Stage (2006‐2010)						
Localized	56 680	44.54	238431.26			
Regional	32 880	25.84	136644.17			
Distant	21 096	16.58	88597.90			
Unknown	16 590	13.04	65218.14			

SD, standard deviation.

### 3.2 Age‐Standardized IRs of first primary cancers and SPCs

Of 565 877 person‐years of follow‐up, 2222 patients developed SPCs. The most common site of SPC in female cancer survivors was the thyroid, which represented 32.76% of all SPCs, followed by cancers of the colorectum (11.16%), lung (10.40%), stomach (7.61%), and breast (5.67%) (Table 2).

**Table 2:** Age‐standardized IRs of the first and second primary cancers

Site	All		Body mass index <25		Body mass index ≥25	
	First cancer	Second cancer	First cancer	Second cancer	First cancer	Second cancer
All cancers, No.	146 377	2222	94 899	1399	51 478	823
Age‐standardized IR	224.77	453.89^e^	221.34	437.87^e^	232.72	577.31^e^
Stomach, No.	23 653	169	15 447	113	8206	56
Age‐standardized IR	36.48	26.44^e^	36.84	27.29^e^	35.74	40.9^e^
Colorectal, No.	22 649	248	13 923	147	8726	101
Age‐standardized IR	33.73	40.71^e^	32.6	38.18^e^	35.89	57.72^e^
Liver, No.	8295	102	4965	63	3330	39
Age‐standardized IR	12.23	15.22^e^	11.76	14.21^e^	13.24	23.72^a^
Pancreas, No.	4497	60	2715	37	1782	23
Age‐standardized IR	7.05	6.53^e^	6.85	6.61^e^	7.58	6.96^a^
Lung, No.	11 786	231	7799	163	3987	68
Age‐standardized IR	18.24	41.08^e^	19.11	44.56^e^	16.55	31^e^
Breast, No.	30 349	126	21 100	80	9249	46
Age‐standardized IR	48.18	26.19^e^	47.21	24.94^e^	49.04	31.19^e^
Uterine cervix, No.	6925	46	4634	24	2291	22
Age‐standardized IR	11.39	7.64^e^	11.14	6.46^e^	11.83	9.26^d^
Thyroid, No.		728		454		274
Age‐standardized IR		192.64		180.62		265.74
Lymphoma, No.	3886	41	2553	21	1333	20
Age‐standardized IR	6.15	4.86^a^	6.1	4.28^e^	6.14	5.75^e^
Ovary, No.	4113	43	2746	23	1367	20
Age‐standardized IR	6.61	8.34^e^	6.44	7.51^e^	7.04	8.77^e^
Oral cavity and pharynx/Esophagus/larynx, No.	1894	38	1265	23	629	15
Age standardized IR	3.05	4.67^e^	3.08	4.82^e^	3.18	4.44^e^
Gallbladder and other biliary, No.	4994	50	2926	32	2068	18
Age‐standardized IR	7.93	6.26^e^	7.44	5.6^e^	8.98	11.54^e^
Uterine corpus, No.	3587	49	2075	31	1512	18
Age‐standardized IR	5.17	11.93^e^	4.36	11.88^e^	8.16	8.02^e^
Kidney, No.	2314	37	1367	24	947	13
Age‐standardized IR	3.44	5.66^e^	3.13	5.78^e^	4.23	5.07^e^
Urinary bladder, No.	1421	23	883	18	538	5
Age‐standardized IR	2.25	4.14^e^	2.21	4.83^e^	2.34	1.52^e^
Leukemia, No.	2144	29	1438	21	706	8
Age‐standardized IR	3.64	6.37^e^	3.57	6.08^e^	4	9.76^e^
Others, No.	13 870	202	9063	125	4807	77
Age‐standardized IR	22.42	48.65^e^	22.57	47.61^e^	22.23	59.82^e^
Obesity‐related, No.^b^	29 786	351	18 228	212	11 558	139
Age‐standardized IR	36.21	49.38^e^	34.86	46.83^e^	39.22	67.37^e^
Obesity‐related female, No.^c^	64 247	520	42 073	315	22 174	205
Age‐standardized IR	91.02	84.35^e^	88.91	79.77^e^	94.63	107.85^e^

IR of cancer per 100 000 person‐years. No. indicates the number of patients with first cancer and SPC.

IR, incidence rate.

a Not significant.

b Includes colorectal, kidney, pancreatic, and esophageal cancers.

c Includes colorectal, kidney, pancreatic, esophageal, breast, and urinary bladder cancers.

d *P *<* *0.05

e *P *<* *0.001

The overall age‐standardized IR of SPCs was 453.9 occurrences per 100 000 person‐years, more than 2.02 times the risk of first primary cancers (224.8 per 100 000 person‐years; *P *<* *0.001) (Table 2). In obese women (BMI ≥ 25 kg/m^2^), the age‐standardized IR of SPCs was approximately 2.5 times greater than that of the first primary cancers (577.31 vs 232.72 per 100 000 person‐years; *P *<* *0.001). In addition, the age‐standardized IR of obesity‐related cancers was also higher for SPCs (67.37 per 100 000 person‐years) than for first primary cancer (39.22 per 100 000 person‐years; *P *<* *0.001) in the obese population.

### 3.3 Associations between prediagnosis BMI and SPCs

There were positive linear trends in the associations between prediagnosis BMI and overall risk of SPC, thyroid, colorectal, ovary, obesity‐related, and obesity‐related female cancers (*P*_trend* *_< 0.05; Table 3). Compared with normal BMI patients, overweight cancer survivors had higher risk of overall SPC (aHR, 1.14; 95% CI, 1.01‐1.29), pancreas (aHR, 2.21; 95% CI, 1.01‐4.85), kidney (aHR, 2.70; 95% CI, 1.08‐6.74), and obesity‐related (aHR, 1.57; 95% CI, 1.16‐2.14) cancers. Obese female cancer survivors had a higher risk of overall SPC (aHR, 1.17; 95% CI, 1.04‐1.32) and thyroid cancer (aHR, 1.41; 95% CI, 1.15‐1.73). In addition, severely obese cancer female survivors showed a higher risk of developing an overall SPC (aHR, 1.32; 95% CI, 1.06‐1.65), colorectal (aHR, 2.31; 95% CI, 1.33‐4.03), thyroid (aHR, 1.79; 95% CI, 1.24‐2.61), and obesity‐related (aHR, 2.03; 95% CI, 1.24‐3.33) cancers. The risk of development of obesity‐related female cancer as SPC also was higher in overweight and obese female cancer survivors (aHR for BMI 23.0‐24.9 kg/m^2^, 1.41; 95% CI, 1.09‐1.82, aHR for BMI 25.0‐29.9 kg/m^2^, 1.35; 95% CI, 1.05‐1.72, and aHR for BMI ≥ 30.0 kg/m^2^, 2.10; 95% CI, 1.40‐3.14, *P*_trend* *_= 0.001). When we computed *P*_*heterogeneity*_ for each type of SPC across the BMI categories, there were no statistically significant differences for the multivariable‐adjusted hazard ratios for SPCs across the BMI categories in female cancer survivors.

**Table 3:** HRs of second primary cancers by prediagnosis BMI in female cancer survivors

Site of SPC	Prediagnosis BMI, kg/m^2^					
	<18.5	18.6‐22.9	23‐24.9	25‐29.9	≥30	*P*_trend_ a
All, No.	56	772	571	719	104	
Age‐adjusted HR	0.82	1	1.13	1.14	1.24	< 0.001
95% CI	0.63‐1.08		1.01‐1.26	1.03‐1.27	1.01‐1.53	
Multivariable‐adjusted HR	0.92	1	1.14	1.17	1.32	0.001
95% CI^b^	0.69‐1.23		1.01‐1.29	1.04‐1.31	1.06‐1.65	
Stomach, No.	7	58	48	50	6	
Age‐adjusted HR	1.3	1	1.26	1.04	0.95	0.860
95% CI	0.60‐2.85		0.86‐1.84	0.71‐1.52	0.41‐2.20	
Multivariable‐adjusted HR	1.69	1	1.31	0.97	0.64	0.263
95% CI^b^	0.76‐3.75		0.86‐1.99	0.63‐1.49	0.23‐1.81	
Colorectal, No.	5	75	67	84	17	
Age‐adjusted HR	0.7	1	1.35	1.33	2.04	0.003
95% CI	0.28‐1.73		0.97‐1.88	0.98‐1.82	1.21‐3.46	
Multivariable‐adjusted HR	0.97	1	1.37	1.23	2.31	0.018
95% CI^b^	0.39‐2.42		0.95‐1.97	0.86‐1.76	1.33‐4.03	
Liver, No.	4	33	26	33	6	
Age‐adjusted HR	1.22	1	1.18	1.17	1.61	0.390
95% CI	0.43‐3.46		0.71‐1.98	0.72‐1.90	0.67‐3.84	
Multivariable‐adjusted HR	0.99	1	1.15	1.1	1.51	0.489
95% CI^b^	0.30‐3.26		0.66‐1.98	0.65‐1.86	0.57‐3.95	
Pancreas, No.	2	16	19	21	2	
Age‐adjusted HR	1.22	1	1.77	1.51	1.09	0.419
95% CI	0.28‐5.30		0.91‐3.45	0.79‐2.90	0.25‐4.76	
Multivariable‐adjusted HR	1.02	1	2.21	1.85	1.32	0.298
95% CI^b^	0.13‐8.06		1.01‐4.85	0.85‐4.01	0.28‐6.15	
Lung, No.	2	103	58	63	5	
Age‐adjusted HR	0.21	1	0.86	0.74	0.44	0.168
95% CI	0.05‐0.85		0.62‐1.18	0.54‐1.01	0.18‐1.09	
Multivariable‐adjusted HR	0.27	1	0.91	0.73	0.45	0.205
95% CI^b^	0.07‐1.09		0.64‐1.29	0.51‐1.05	0.17‐1.25	
Breast, No.	5	40	35	39	7	
Age‐adjusted HR	1.5	1	1.35	1.23	1.66	0.336
95% CI	0.59‐3.80		0.86‐2.12	0.80‐1.91	0.74‐3.70	
Multivariable‐adjusted HR	1.58	1	1.2	1.36	2.3	0.138
95% CI^b^	0.62‐4.06		0.71‐2.02	0.83‐2.22	1.00‐5.29	
Uterine cervix, No.	0	17	7	20	2	
Age‐adjusted HR	0	1	0.63	1.43	1.08	0.161
95% CI	0		0.26‐1.51	0.75‐2.73	0.25‐4.69	
Multivariable‐adjusted HR	0	1	0.53	1.41	0.67	0.328
95% CI^b^	0		0.19‐1.47	0.68‐2.94	0.09‐5.15	
Thyroid, No	14	255	185	235	39	
Age‐adjusted HR	0.69	1	1.12	1.18	1.46	0.003
95% CI	0.40‐1.19		0.93‐1.36	0.99‐1.41	1.04‐2.04	
Multivariable‐adjusted HR	0.61	1	1.23	1.41	1.8	<0.001
95% CI^b^	0.34‐1.09		0.99‐1.53	1.15‐1.73	1.24‐2.61	
Lymphoma, No.	0	12	9	18	2	
Age‐adjusted HR	0	1	1.15	1.85	1.56	0.057
95% CI	0		0.48‐2.72	0.89‐3.85	0.35‐6.99	
Multivariable‐adjusted HR	0	1	1.05	1.64	1.8	0.103
95% CI^b^	0		0.42‐2.63	0.74‐3.65	0.39‐8.36	
Ovary, No.	1	15	7	17	3	
Age‐adjusted HR	0.79	1	0.72	1.42	1.87	0.169
95% CI	0.10‐5.99		0.29‐1.76	0.71‐2.84	0.54‐6.46	
Multivariable‐adjusted HR	1.2	1	0.92	2.09	2.77	0.041
95% CI^b^	0.15‐9.41		0.33‐2.55	0.93‐4.70	0.73‐10.43	
Oral cavity and pharynx/Esophagus/larynx, No.	0	17	6	12	3	
Age‐adjusted HR	0	1	0.54	0.85	1.6	0.488
95% CI	0		0.21‐1.36	0.41‐1.78	0.47‐5.45	
Multivariable‐adjusted HR	0	1	0.47	0.75	1.86	0.552
95% CI^b^	0		0.17‐1.31	0.33‐1.71	0.52‐6.62	
Gallbladder and other biliary, No.	1	16	15	16	2	
Age‐adjusted HR	0.6	1	1.4	1.14	1.08	0.728
95% CI	0.08‐4.54		0.69‐2.83	0.57‐2.29	0.25‐4.71	
Multivariable‐adjusted HR	0.95	1	1.36	1.3	1.31	0.538
95% CI^b^	0.12‐7.38		0.61‐3.05	0.59‐2.83	0.29‐6.01	
Uterine corpus, No.	1	14	16	17	1	
Age‐adjusted HR	0.91	1	1.77	1.56	0.69	0.465
95% CI	0.12‐6.91		0.86‐3.62	0.77‐3.16	0.09‐5.23	
Multivariable‐adjusted HR	0.88	1	1.77	2.09	1.19	0.134
95% CI^b^	0.11‐6.81		0.79‐3.99	0.95‐4.59	0.15‐9.34	
Kidney, No.	1	8	15	11	2	
Age‐adjusted HR	1.36	1	2.85	1.67	2.29	0.279
95% CI	0.17‐10.90		1.21‐6.73	0.67‐4.15	0.49‐10.79	
Multivariable‐adjusted HR	1.71	1	2.7	1.28	1.98	0.692
95% CI^b^	0.21‐13.98		1.08‐6.74	0.47‐3.52	0.40‐9.84	
Urinary bladder, No.	1	11	6	5	0	
Age‐adjusted HR	0.88	1	0.82	0.52	0	0.136
95% CI	0.11‐6.84		0.30‐2.21	0.18‐1.51	0‐.	
Multivariable‐adjusted HR	1.47	1	0.8	0.56	0	0.131
95% CI^b^	0.18‐11.95		0.26‐2.46	0.18‐1.77	0‐.	
Leukemia, No.	1	13	7	7	1	
Age‐adjusted HR	0.89	1	0.82	0.67	0.72	0.447
95% CI	0.12‐6.78		0.33‐2.07	0.27‐1.67	0.10‐5.54	
Multivariable‐adjusted HR	1.22	1	0.8	0.72	1	0.583
95% CI^b^	0.16‐9.50		0.29‐2.19	0.257‐2.00	0.13‐8.00	
Obesity‐related, No.^c^	8	101	103	118	21	
Age‐adjusted HR	0.82	1	1.54	1.39	1.87	0.0014
95% CI	0.40‐1.69		1.17‐2.03	1.06‐1.81	1.17‐2.99	
Multivariable‐adjusted HR	1.02	1	1.57	1.3	2.03	0.013
95% CI^b^	0.47‐2.21		1.16‐2.14	0.96‐1.76	1.24‐3.33	
Obesity‐related female, No.^d^	14	156	154	174	31	
Age‐adjusted HR	0.97	1	1.41	1.35	1.81	0.001
95% CI	0.56‐1.68		1.13‐1.77	1.09‐1.67	1.23‐2.67	
Multivariable‐adjusted HR	1.22	1	1.41	1.35	2.10	0.001
95% CI^b^	0.69‐2.17		1.09‐1.82	1.05‐1.72	1.40‐3.14	

No. indicates the number of patients with SPC.

BMI, body mass index; CI, confidence interval; HR, hazard ratio; SPC, secondary primary cancer.

a Tests for trends were performed by assigning a median value for the BMI and treating the new variable as a continuous term in the models.

b The multivariable hazard ratio model used Cox proportional analysis and adjusted age (continuous variable), smoking status, alcohol consumption frequency, physical activity times, fasting serum glucose level, fasting serum cholesterol level, comorbidity, and average insurance premium per month.

c Includes colorectal, kidney, pancreatic, and esophageal cancers.

d Includes colorectal, kidney, pancreatic, esophageal, breast, and urinary bladder cancers.

The BMI‐SPC risk association was statistically significant in female cancer survivors without smoking history (*P*_trend _= 0.001), with a localized stage in the first primary cancer (*P*_trend _= 0.014) and an obesity‐related cancer as the first cancer (*P*_trend* *_= 0.036, Table 4). In addition, the BMI‐SPC risk association was higher among cancer survivors living in metropolitan areas (aHR for BMI ≥ 30.0 kg/m^2^, 2.05; 95% CI, 1.22‐3.46; *P*_trend* *_= 0.001).

**Table 4:** Stratified, multivariable analysis of risk of any SPC by prediagnosis BMI in female cancer survivors

	Prediagnosis BMI, kg/m^2^					
	<18.5	18.6‐22.9	23‐24.9	25‐29.9	≥30	*P*_trend_ a
Smoking status						
Never‐smoker, No.	53	696	524	681	99	
Multivariable‐adjusted HR	0.99	1	1.14	1.19	1.36	0.001
95% CI^b^	0.74‐1.32		1.00‐1.29	1.05‐1.34	1.08‐1.70	
Ever‐smoker, No.	2	49	30	18	2	
Multivariable‐adjusted HR	0.34	1	1.28	0.81	0.65	0.976
95% CI^b^	0.08‐1.41		0.77‐2.15	0.45‐1.44	0.16‐2.76	
Age at first cancer diagnosis						
Age < 60 y, No.	30	470	272	338	47	
Multivariable‐adjusted HR	0.95	1	1.00	1.12	1.18	0.119
95% CI^b^	0.64‐1.42		0.85‐1.18	0.95‐1.32	0.84‐1.67	
Age ≥ 60 y, No.	26	302	299	381	57	
Multivariable‐adjusted HR	1.10	1	1.19	1.103	1.29	0.186
95% CI^b^	0.73‐1.67		1.00 ‐1.422	0.93‐1.30	0.96‐1.74	
Stage						
Localized, No.	17	288	204	292	36	
Multivariable‐adjusted HR	0.75	1	1.09	1.21	1.10	0.014
95% CI^b^	0.47‐1.19		0.91‐1.30	1.03‐1.43	0.77‐1.56	
Regional, No.	16	134	119	124	18	
Multivariable‐adjusted HR	1.71	1	1.494	1.22	1.54	0.208
95% CI^b^	1.03‐2.84		1.16‐1.92	0.95‐1.57	0.98‐2.41	
Distance, No.	6	66	49	50	9	
Multivariable‐adjusted HR	0.74	1	1.05	1.04	1.43	0.681
95% CI^b^	0.30‐1.83		0.72‐1.54	0.72‐1.51	0.70‐2.92	
Place of residence						
Metropolitan area, No.	11	110	123	133	18	
Multivariable‐adjusted HR	1.33	1	1.76	1.63	2.05	0.001
95% CI^b^	0.67‐2.66		1.30‐2.37	1.21‐2.20	1.22‐3.46	
City, No.	9	224	142	168	30	
Multivariable‐adjusted HR	0.543	1	0.957	0.982	1.573	0.167
95% CI^b^	0.27‐1.10		0.76‐1.21	0.78‐1.23	1.05‐2.37	
Rural county, No.	36	438	306	418	56	
Multivariable‐adjusted HR	1.01	1	1.08	1.14	1.07	0.126
95% CI^b^	0.71‐1.44		0.92‐1.27	0.98‐1.33	0.78‐1.46	
Average insurance premium per month						
1st quarter, No.	12	160	135	159	26	
Multivariable‐adjusted HR	0.90	1	1.27	1.2	1.46	0.039
95% CI^b^	0.50‐1.62		1.00‐1.62	0.95‐1.52	0.95‐2.25	
2nd quarter, No.	17	164	107	172	29	
Multivariable‐adjusted HR	1.06	1	1.02	1.20	1.44	0.060
95% CI^b^	0.63‐1.77		0.79‐1.31	0.95‐1.51	0.95‐2.19	
3rd quarter, No.	11	175	122	134	16	
Multivariable‐adjusted HR	0.80	1	1.09	0.97	0.92	0.963
95% CI^b^	0.43‐1.47		0.85‐1.39	0.76‐1.24	0.55‐1.56	
4th quarter, No.	13	170	123	168	22	
Multivariable‐adjusted HR	1.03	1	1.10	1.24	1.37	0.040
95% CI^b^	0.57‐1.86		0.86‐1.39	0.99‐1.56	0.88‐2.19	
Presence of disability						
No disability, No.	53	750	542	664	87	
Multivariable‐adjusted HR	0.90	1	1.13	1.14	1.22	0.009
95% CI^b^	0.67‐1.21		1.00‐1.28	1.01‐1.28	0.96‐1.56	
Disability, No.	3	22	29	55	17	
Multivariable‐adjusted HR	1.86	1	1.50	1.950	2.71	0.006
95% CI^b^	0.54‐6.36		0.81‐2.79	1.13‐3.38	1.34‐5.50	
Cancer survivors whose first cancer was obesity‐related cancer, No.^c^	11	130	128	154	25	
Multivariable‐adjusted HR	1.33	1	1.36	1.34	1.57	0.036
95% CI^b^	0.71‐2.49		1.03‐1.79	1.03‐1.74	0.97‐2.54	
Cancer survivors whose first cancer was obesity‐related cancer, No.^d^	31	370	282	334	54	
Multivariable‐adjusted HR	1.29		1.16	1.13	1.34	0.148
95% CI^b^	0.88‐1.90		0.97‐1.34	0.95‐1.34	0.97‐1.85	

No. indicates the number of patients with SPC.

BMI, body mass index; CI, confidence interval; HR, hazard ratio; SPC, secondary primary cancer.

a Tests for trends were performed by assigning a median value for the BMI and treating the new variable as a continuous term in the models.

b The multivariable hazard ratio model used Cox proportional analysis and adjusted age (continuous variable), smoking status, alcohol consumption frequency, physical activity times, fasting serum glucose level, fasting serum cholesterol level, comorbidity, and average insurance premium per month.

c Includes colorectal, kidney, pancreatic, and esophageal cancers.

d Includes colorectal, kidney, pancreatic, esophageal, breast, and endometrial cancers.

In analyses of SPC risk that allowed for death as a competing risk, subdistribution HRs for SPC were statistically significant in overweight (aHR, 1.06; 95% CI, 1.03‐1.29) and obese (aHR, 1.09; 95% CI, 1.06‐1.12) female cancer survivors (*P*_trend* *_< 0.001). Five‐year cumulative mortality among female cancer survivors was higher in the overweight (65.27%), obese (63.78%), and severely obese (62.90%) female cancer survivors than in the normal (66.50%) and underweight (54.87%) groups. Compared to groups with normal BMI, the severely obese survivors had a subdistribution HR for death of 1.13 (95% CI, 1.11‐1.16; *P*_trend* *_< 0.001).

### 3.4 Comparison of the association between BMI and risk of first cancer in the general population

We compared the magnitude of the association between obesity and risk of SPC among female cancer survivors with the association between BMI and risk of first cancer in cancer‐free general population (Table 5). We included 565 877 person‐years of follow‐up in our analysis, and we documented 146 377 patients with primary cancers. Overall, the BMI‐cancer associations between BMI and first cancer risk were similar to associations between BMI and SPC risk in female cancer survivors. In the severely obese category, the aHRs for SPCs in female cancer survivors (aHR, 1.32; 95% CI, 1.06‐1.65) were similar to those with first cancers in cancer‐free general population (aHR, 1.20; 95% CI, 1.17‐1.24, *P*_heterogeneity* *_= 0.403). In the obese category, the magnitude of the BMI‐SPC risk association was similar to the association with the first cancers (aHR, 1.17; 95% CI, 1.04‐1.31 vs aHR, 1.12; 95% CI, 1.10‐1.13, *P*_heterogeneity* *_= 0.461).

**Table 5:** HRs of first cancer by BMI in female cohort participants

Site of first cancer	Prediagnosis BMI, kg/m^2^					
	<18.5	18.6‐22.9	23‐24.9	25‐29.9	≥30	*P*_trend_ a
All, No.	4958	54 277	35 664	45 268	6 210	
Age‐adjusted HR	0.67	1	1.34	1.46	1.55	<0.0001
95% CI	0.65‐0.69		1.32‐1.35	1.44‐1.48	1.51‐1.59	
Multivariable‐adjusted HR	0.83	1	1.08	1.12	1.20	<0.0001
95% CI^b^	0.81‐0.86		1.06‐1.09	1.10‐1.13	1.17‐1.24	
Stomach, No.	853	8781	5813	7297	909	
Age‐adjusted HR	0.707	1	1.312	1.397	1.35	<0.0001
95% CI	0.66‐0.76		1.27‐1.36	1.35‐1.44	1.26‐1.45	
Multivariable‐adjusted HR	0.89	1	1.04	1.05	1.03	<0.0001
95% CI^b^	0.82‐0.95		1.01‐1.08	1.02‐1.08	0.96‐1.10	
Colorectal, No.	596	7629	5698	7649	1077	
Age‐adjusted HR	0.57	1	1.48	1.68	1.83	<0.0001
95% CI	0.53‐0.62		1.43‐1.53	1.63‐1.74	1.72‐1.95	
Multivariable‐adjusted HR	0.72	1	1.14	1.21	1.34	<0.0001
95% CI^b^	0.66‐0.79		1.10‐1.18	1.17‐1.25	1.26‐1.43	
Liver, No.	235	2685	2045	2888	442	
Age‐adjusted HR	0.65	1	1.49	1.77	2.09	<0.0001
95% CI	0.57‐0.74		1.40‐1.58	1.67‐1.86	1.89‐2.31	
Multivariable‐adjusted HR	0.72	1	1.24	1.41	1.77	<0.0001
95% CI^b^	0.63‐0.83		1.17‐1.32	1.33‐1.49	1.59‐1.96	
Pancreas, No.	137	1424	1154	1574	208	
Age‐adjusted HR	0.73	1	1.48	1.63	1.67	<0.0001
95% CI	0.61‐0.86		1.37‐1.60	1.52‐1.75	1.44‐1.93	
Multivariable‐adjusted HR	0.82	1	1.20	1.25	1.28	<0.0001
95% CI^b^	0.69‐0.98		1.11‐1.30	1.16‐1.35	1.10‐1.48	
Lung, No.	518	4419	2862	3578	409	
Age‐adjusted HR	0.87	1	1.214	1.245	1.11	0.002
95% CI	0.79‐0.95		1.16‐1.27	1.19‐1.30	1.00‐1.23	
Multivariable‐adjusted HR	0.96	1	0.99	0.98	0.88	0.103
95% CI^b^	0.88‐1.06		0.95‐1.04	0.93‐1.02	0.80‐0.98	
Breast, No.	903	12 943	7254	8089	1160	
Age‐adjusted HR	0.50	1	1.27	1.33	1.47	<0.0001
95% CI	0.47‐0.54		1.23‐1.31	1.29‐1.36	1.38‐1.56	
Multivariable‐adjusted HR	0.54	1	1.17	1.17	1.33	<0.0001
95% CI^b^	0.503‐0.578		1.13‐1.20	1.14‐1.21	1.25‐1.41	
Uterine cervix, No.	299	2746	1589	2035	256	
Age‐adjusted HR	0.79	1	1.24	1.42	1.38	<0.0001
95% CI	0.71‐0.89		1.17‐1.32	1.34‐1.51	1.21‐1.57	
Multivariable‐adjusted HR	0.93	1	1.07	1.16	1.11	<0.0001
95% CI^b^	0.83‐1.06		1.00‐1.14	1.09‐1.24	0.97‐1.27	
Lymphoma, No.	140	1458	955	1182	151	
Age‐adjusted HR	0.71	1	1.35	1.42	1.45	0.001
95% CI	0.60‐0.84		1.24‐1.46	1.34‐1.57	1.23‐1.71	
Multivariable‐adjusted HR	0.89	1	1.10	1.12	1.15	0.001
95% CI^b^	0.74‐1.06		1.01‐1.20	1.03‐1.22	0.97‐1.37	
Ovary, No.	160	1551	1035	1196	171	
Age‐adjusted HR	0.74	1	1.45	1.52	1.67	<0.0001
95% CI	0.63‐0.87		1.34‐1.57	1.40‐1.64	1.43‐1.96	
Multivariable‐adjusted HR	0.87	1	1.18	1.18	1.35	<0.0001
95% CI^b^	0.74‐1.03		1.09‐1.27	1.092‐1.28	1.15‐1.59	
Oral cavity and pharynx/Esophagus/larynx, No.	94	743	428	547	82	
Age‐adjusted HR	0.92	1	1.16	1.27	1.48	0.536
95% CI	0.75‐1.14		1.03‐1.31	1.14‐1.42	1.18‐1.86	
Multivariable‐adjusted HR	1.08	1	0.94	1.00	1.12	0.896
95% CI^b^	0.86‐1.35		0.83‐1.07	0.89‐1.13	0.88‐1.42	
Gallbladder and other biliary, No.	144	1547	1235	1819	249	
Age‐adjusted HR	0.70	1	1.46	1.74	1.86	<0.0001
95% CI	0.59‐0.83		1.36‐1.58	1.63‐1.87	1.63‐2.13	
Multivariable‐adjusted HR	0.78	1	1.18	1.36	1.48	<0.0001
95% CI^b^	0.66‐0.93		1.09‐1.27	1.26‐1.46	1.29‐1.70	
Uterine corpus, No.	77	1146	852	1225	287	
Age‐adjusted HR	0.50	1	1.68	2.24	4.060	<0.0001
95% CI	0.40‐0.62		1.53‐1.83	2.07‐2.43	3.57‐4.62	
Multivariable‐adjusted HR	0.51	1	1.44	1.81	3.37	<0.0001
95% CI^b^	0.40‐0.65		1.31‐1.59	1.66‐1.98	2.94‐3.86	
Kidney, No.	61	740	566	826	121	
Age‐adjusted HR	0.61	1	1.59	2.03	2.30	<0.0001
95% CI	0.48‐0.79		1.42‐1.77	1.84‐2.25	1.90‐2.78	
Multivariable‐adjusted HR	0.70	1	1.24	1.48	1.65	<0.0001
95% CI^b^	0.52‐0.93		1.11‐1.39	1.33‐1.64	1.346‐2.02	
Urinary bladder, No.	50	486	347	463	75	
Age‐adjusted HR	0.76	1	1.30	1.40	1.75	0.001
95% CI	0.57‐1.01		1.13‐1.50	1.23‐1.59	1.37‐2.24	
Multivariable‐adjusted HR	0.85	1	1.06	1.08	1.42	0.005
95% CI^b^	0.63‐1.14		0.92‐1.22	0.95‐1.24	1.10‐1.82	
Leukemia, No.	109	806	523	619	87	
Age‐adjusted HR	0.97	1	1.34	1.38	1.51	0.060
95% CI	0.80‐1.19		1.20‐1.49	1.24‐1.54	1.21‐1.88	
Multivariable‐adjusted HR	1.14	1	1.15	1.18	1.26	0.009
95% CI^b^	0.93‐1.40		1.02‐1.29	1.05‐1.32	1.00‐1.59	
Others, No.	582	5173	3308	4281	526	
Age‐adjusted HR	0.82	1	1.23	1.33	1.27	<0.0001
95% CI	0.76‐0.90		1.18‐1.29	1.28‐1.39	1.16‐1.38	
Multivariable‐adjusted HR	1.00	1	1.01	1.04	1.02	0.093
95% CI^b^	0.91‐1.09		0.96‐1.06	1.00‐1.09	0.93‐1.12	

No. indicates the number of patients with first cancer.

BMI, body mass index; CI, confidence interval; HR, hazard ratio.

a Tests for trends were performed by assigning a median value for the BMI and treating the new variable as a continuous term in the models.

b The multivariable hazard ratio model used Cox proportional analysis and adjusted age (continuous variable), smoking status, alcohol consumption frequency, physical activity times, fasting serum glucose level, fasting serum cholesterol level, comorbidity, and average insurance premium per month.

## 4 DISCUSSION

Using the Korean female cancer survivor cohort, we demonstrated that obese female cancer survivors before first primary cancer diagnosis had a higher risk of subsequent SPC and increased risk of thyroid, colorectal, ovary, obesity‐related, and obesity‐related female cancers. In addition, the BMI‐SPC risk association was statistically significant in female cancer survivors without smoking history, with a localized first primary cancer, and who lived in a metropolitan area. However, there was no difference in the magnitude of the association between obesity and SPC risk in female cancer survivors compared to that of primary cancer risk in cancer‐free general population.

Previous studies reported that obesity is a risk factor for primary cancers, such as breast, colorectal, liver, and kidney in the general population,^17^, ^18^, ^19^, ^34^ which is consistent with our finding that obesity increased the risk of first primary cancer. In this study, we could find the significant dose‐dependent relationships between prediagnosis BMI and overall SPCs including obesity‐related, obesity‐related female, and several individual SPCs. Previous studies have identified obese patients have experienced higher recurrences in colorectal, pancreatic, prostate, and breast cancers.^35^, ^36^, ^37^, ^38^ In a cohort of over 10 000 breast cancer survivors, the HR of SPCs in overweight women was 2.23 (95% CI, 1.23‐4.05) for endometrial cancer, 1.67 (95% CI, 0.99‐2.82) for colorectal cancer, and 0.80 (95% CI, 0.28‐2.29) for ovarian cancer.^39^ In a meta‐analysis of prospective studies for excess body weight and SPC risk after breast cancer, obesity was associated with significantly increased risk of SPC of the contralateral breast (relative risk (RR), 1.37; 95% CI, 1.20‐1.57), breast (RR, 1.40; 95% CI, 1.24‐1.58), endometrium (RR, 1.96; 95% CI, 1.43‐2.70), and colorectum (RR, 1.89; 95% CI, 1.28‐2.79).^23^ In a pooled analysis of five prospective cohort studies for colorectal cancer survivors, overweight and obese survivors had an increased risk of a second obesity‐associated cancer (aHR, 1.39; 95% CI, 1.01‐1.92; aHR, 1.47; 95% CI, 1.02‐2.12, respectively) compared to survivors with normal prediagnosis BMI.^24^ In a prospective cohort study of a large Korean population, the incidence of thyroid cancer was positively associated with higher BMI in women younger than 50 years of age (HR, 1.57 for BMI ≥ 25.0 Kg/m^2^; 95% CI, 1.28‐1.92).^40^

The mechanisms by which obesity confers an increased risk of first primary cancer or SPCs are likely to be similar. The positive association between obesity and risk of SPC in female cancer survivors could be, in a large part, explained by increased circulating estrogen, other circulating hormones, or other growth factors, or by a low‐grade chronic inflammatory state.^21^, ^41^, ^42^ Furthermore, it could be explained by additional genetic susceptibility or the carcinogenic effects due to cancer treatment. Interestingly, our study showed that higher BMI was more in female cancer survivors than in general population and the association between obesity and SPC risk among female cancer survivors was similar to that of first primary cancer risk in cancer‐free general population. These findings are different from previous results that the strength of the BMI‐cancer association was slightly stronger in male cancer survivors than in the general population.^27^ These suggest that association between obesity and risk of SPC could be different by gender. In addition, our study showed that the BMI‐SPC risk association was statistically significant in female cancer survivors without smoking history and those with a localized first primary cancer. This suggests that obesity could affect SPC in female cancer survivors who have healthier lifestyle or expect longer life expectancy.

In subgroup analyses, the HR for SPC according to prediagnosis BMI was significantly higher in female cancer survivors who did not have smoking history or who had localized primary cancer. Smoking history would be a confounding factor in this study. In addition, it could be explained by that the cancer survivors have a relatively healthier lifestyle or because early‐stage cancers might confer a greater chance for long‐term survival, these findings could inform planning for long‐term cancer survivorship programs, such as including early SPC detection.

Strengths of this study are that we could use the large‐scaled, prospective cohort with approximate 150 000 female cancer survivors, including detailed information of prediagnosis behavioral risk factors. Our study design reduced recall bias, which could be a limitation in retrospective studies of cancer survivors. Specifically, height and weight, the main variables in our study, were measured by trained persons, not based on self‐report. To our knowledge, this is the first large cohort study of female cancer survivors to show that prediagnosis BMI might affect the risk of the subsequent overall, obesity‐related, and individual SPCs.

Because this is parallel study with the previous study for male cancer survivors,^27^ we have same limitations with the previous report. First, we could not consider the effects of cancer treatment on SPCs because of lack of detailed information about cancer treatment. This study has the possibility of selection bias because we analyzed only the population with height and weight data from the NHIS. This cohort has some possibility for SPC misclassification. Therefore, we performed sensitivity analyses for adjusting this possibility, excluding patients with SPC that diagnosed within 2 years after the first cancer diagnosis, and showed that the overall trends remained similar. In addition, we excluded thyroid cancer as primary cancer because thyroid cancer in Korea has been epidemically increased because of screening and considered as overdiagnosis.^43^ In this study, we could not evaluate the effect on the changes in BMI for female survivors because of the lack of data. So, we need to consider this limitation and try to perform the further study on the changes in BMI and SPC in future.

This study demonstrated that prediagnosis obesity increased risk of overall and individual SPCs in female cancer survivors. However, the magnitude of the association between obesity and SPC risk among female cancer survivors was similar to that of first primary cancer risk in the overall cohort. These findings suggest that lifestyle modification for weight reduction should be encouraged for prevention of primary cancer and SPCs, and individualized surveillance should be supported for obese female cancer survivors. In future, more studies should be performed to explain the different BMI‐cancer associations by gender.

## CONFLICT OF INTEREST

The authors have declared no conflicts of interest.
